# Population genetics of the critically endangered three-striped turtle, *Batagur dhongoka*, from the Ganga river system using mitochondrial DNA and microsatellite analysis

**DOI:** 10.1038/s41598-024-54816-0

**Published:** 2024-03-11

**Authors:** Ajit Kumar, Ashish Kumar Panda, Aftab Alam Usmani, Prabhaker Yadav, Anshu Panwar, Ruchi Badola, Syed Ainul Hussain, Sandeep Kumar Gupta

**Affiliations:** 1 Wildlife Institute of India, P.O. Box # 18, Chandrabani, Dehra Dun, Uttarakhand 248001 India; 2 Present Address: Department of System Biology, Centre for Biomedical Research, SGPGIMS Campus, Lucknow, India

**Keywords:** Genetic diversity, Heterozygosity, mtDNA, Microsatellite, Population structure, Structural variation, Molecular ecology, DNA sequencing, Haplotypes

## Abstract

The three-striped roofed (*Batagur dhongoka*) is a semi-aquatic turtle that belongs to family Geoemydidae. Due to anthropogenic pressure, it has been facing an intense decline of over 80% in its distribution range in the past 50 years. It is considered as 'Critically Endangered' so effective conservation strategies are needed to protect the species by determining their genetic diversity and population genetic structure. This study investigates the genetic diversity, population structure and demographic pattern of *B. dhongoka* from two Turtle Rescue and Rehabilitation Centre established near Ganga river using mitochondrial cytochrome *b* (Cyt *b*: 1140 bp) ; control region (CR: 451 bp) and ten nuclear microsatellite loci. mtDNA results show low levels of nucleotide diversity (π = 0.0022) in *B. dhongoka* haplotypes and provide evidence for a low substitution rate. The demographic pattern estimated by the Bayesian skyline plot (BSP) analysis indicates historical stability followed by growth in the effective population size, with a recent reduction in population size from ~ 2 thousand years ago. The microsatellite findings show a moderate level of observed heterozygosity (Ho: 0.49). Bayesian-based clustering analysis revealed weak genetic structures in *B. dhongoka* and presence of admixed assignations suggesting close genetic relationships. These findings shed light on *B. dhongoka's* genetic status and underline the necessity of comprehensive rehabilitation and relocation programs and conservation and management techniques to ensure the species' long-term survival. In order to ensure the effective protection and conservation of *B. dhongoka*, the Government of India has taken a proactive measure by incorporating it into Schedule *I* of the Wildlife (Protection) Act, 1972, as amended in 2022.

## Introduction

Turtles are the most threatened biota on the earth, with 40% of all extant turtles facing extinction risk^1,2^. All six extant *Batagur* species are categorized as ‘Critically Endangered’ (CR) in the IUCN Red list. The three-striped roofed turtle, *B. dhongoka* is a freshwater turtle confined to northern India (Ganga river basin), Nepal, and Bangladesh^3,4^. Once inhabiting the Ganga and Brahmaputra basin, the *B. dhongoka* populations are now drastically declining throughout its range by over 80%, due to unsustainable human pressure that has deteriorated riverine habitats^5–7^. As a result, the population of *B. dhongoka* has been extirpated from the Gomti River, a tributary of the Ganga, with a major population now restricted to the National Chambal Sanctuary, India^3^.

Consequently, there is a critical need to reevaluate the present distribution of this species^5,6^. Habitat loss and degradation is the principal threat to *B. dhongoka* survival, as it degrades nesting sites, nursery habitats and basking sites and decreases food availability^3^. Additionally, this species is used for subsistence consumption and is involved in some commercial trade, with recent confiscations reported in Malaysia and China^3^. Due to the increasing decline in its native range and a corresponding rise in international seizures, *B. dhongoka* has gained the highest level of protection within its native regions. In 2022, it was included in Schedule *I* of the Indian Wildlife (Protection) Act 1972 and added to Appendix II of CITES.

It is essential to implement extended monitoring efforts, identify sustainable populations, and execute a scientifically guided reintroduction throughout their native habitat to ensure the long-term conservation of *B. dhongoka*. In 2006, the state forest department and Turtle Survival Alliance initiated the Chambal Conservation Project in the National Chambal Sanctuary to increase and conserve the population of *B. dhongoka* and *B. kachuga*^8^. Further, under the National Mission for Clean Ganga project, Wildlife Institute of India, Dehradun, the Turtle Rescue and Rehabilitation Centre (TRRC) at Sarnath, Varanasi was ameliorated and the Ganga Aqualife Rescue and Rehabilitation Centre (GARRC), Narora at Uttar Pradesh was established. Given the challenges and reduction in the population size of freshwater turtles in Ganga river and as part of conservation efforts Turtle Rescue and Rehabilitation Centers play a crucial role in bolstering population numbers and providing valuable support to management initiatives. As part of a conservation project, the Forest Department rescued turtle eggs from the Chambal River and transferred them to the TRRC, Sarnath for hatching^9^. After reaching a certain size, healthy individuals were released into the suitable habitat of the Ganga River. The TRRC also received rescued individuals seized by enforcement agencies from local areas. The GARRC exclusively received rescued individuals, comprising those either injured or accidentally entangled in fishing nets within the local vicinity of the Ganga River. Analyzing a declining population's genetic structure and diversity holds profound implications for its conservation, especially when individuals are chosen to increase the effectiveness of endeavors aimed at successful breeding, rehabilitation, and reintroduction^10^. A decrease in population size can affect long-term evolutionary potential by reducing individual fitness and genetic variation in populations^11^. The eventual strategy to prevent extinction or increase population size is scientifically informed through rescue and rehabilitation, captive breeding, and reintroducing healthy individuals into wild habitats^6^. However, rescue and rehabilitation, captive breeding and reintroduction in the wild have critical complications. Gathering information on the geographic origin of individuals is sometimes difficult and the release of these unknown origin rescue individuals in the wild is often accompanied by high initial losses of released individuals^12,13^. Hence, while a small number of individuals are released locally, genetic monitoring is vital to understand the wider and long-term contemporary genetic diversity for effective management and conservation interventions.

Conservation genetics provides a robust framework for assessing the extent of genetic diversity and population genetic structure of small and declining populations. It plays a vital role in conservation efforts by developing and utilizing comprehensive genetic databases^14^. Until now, only limited information on ecological and genetic aspects of *B. dhongoka* has been available, apart from a comparative mitogenome analysis between *B. kachuga* and *B. dhongoka*^15^. Additionally, the utilization of the genomic approach by Çilingir et al.^16^ on the Burmese roofed turtle (*B. trivittata*) in Myanmar, as well as investigation of the southern river terrapin (*B. affinis*) in Cambodia^17^, provided estimations of wild breeder populations and insights into the present genetic structure. Recent study revealed low genetic diversity and notable genetic differences among the *B. affinis* populations in Peninsular Malaysia^18^.

In this study, we investigate the genetic diversity and population structure of *B. dhongoka* using two mitochondrial DNA (mtDNA) regions and microsatellite DNA markers. Our study focuses on *B. dhongoka* from two Turtle Rescue and Rehabilitation Centers: (1) TRRC at Sarnath, Varanasi, and (2) GARRC at Narora, Uttar Pradesh. Understanding the genetic diversity and population structure of *B. dhongoka* turtles within these Rescue and Rehabilitation centers can have profound impact on rehabilitation and conservation efforts by adopting evidence-based management practices.

## Results

### Genetic diversity

Genetic diversity parameters revealed with mtDNA and microsatellite markers in the *B. dhongoka* from TRRC and GARRC are presented in Table 1. In this study, two mtDNA fragments were obtained from 92 samples; after alignment, 1140 bp of cyt *b* and 451 bp of the control region were generated, producing a concatenated fragment of 1591 bp. All generated sequences were deposited in GenBank (OQ378390–OQ378573). In overall samples, 15 variable sites were observed, thus accounting for only 0.94% of the total sites. All 15 were parsimony-informative sites, comprising 13 transitions and two transversions. We identified 31 haplotypes with an average of 4.59 nucleotide differences (K) in both TRRC and GARRC populations. Among 31 haplotypes, 28 were detected in TRRC with K = 4.55 and six were found in GARRC with K = 3.39, while four haplotypes (H1-H4) are common in both populations. The haplotypes (h) and nucleotide diversity (π) of TRRC were 0.932 ± 0.013 and 0.003 ± 0.00012, respectively and those of GARRC were 0.85 ± 0.074 and 0.002 ± 0.0002, respectively. The overall Hd and π were 0.94 ± 0.012 and 0.0022 ± 0.00014, respectively (Table 1).

**Table 1:** Summary of genetic diversity in *Batagur dhongoka* populations based on concatenated mtDNA (Cyt *b* + CR) and ten microsatellites.

Population/region	Mitochondrial DNA							Microsatellites				
	n	H	Hd	π	K	Tajima’s D*	Fu's F_S_*	Na	Ar	Ho	H_E_	FIS
Turtle Rescue and Rehabilitation Centre (TRRC) Sarnath, Uttar Pradesh, India	80	29	0.93	0.003	4.6	2.04	− 12.3	5	3.84	0.487	0.493	0.018
Ganga Aqualife Rescue and Rehabilitation Centre (GARRC) Narora, , Uttar Pradesh, India	12	6	0.85	0.002	3.4	1.816	0.015	3.4	3.4	0.497	0.5	0.038
Total	92	31	0.94	0.002	4.6	1.56	− 1.86	4.1	3.82	0.495	0.494	0.028

n Number of samples, H haplotype, Hd haplotype diversity, π nucleotide diversity, Na number of alleles, Ar allelic richness, HO observed heterozygosity, HE expected heterozygosity, FIS inbreeding coefficient, *all *P*-values > 0.01 (not significant).

In addition, the selected microsatellite markers showed polymorphic information content with a mean value of 0.476. Loci Maucas18 had the highest allele counts, while Maucas3 showed the lowest. The analysis of microsatellite data did not find evidence of LD. We did not find evidence of a large allele dropout and scoring error in our data. The mean number of alleles (Na) in TRRC and GARRC were 5 and 3.4, respectively, with comparative allelic richness (Ar) in TRRC (Ar = 3.84) and GARRC (Ar = 3.4) with mean Ar = 3.82 (Supplementary table ST1). The observed heterozygosity (Ho) and expected heterozygosity (HE) in TRRC were Ho: 0.487; HE: 0.493; and in GARRC were Ho: 0.497; HE: 0.50 with mean Ho: 0.495; HE: 0.495. The mean inbreeding coefficient (F_IS_) value for both the rescued *B. dhongoka* populations was greater than zero mean F_IS_: 0.028, indicating a heterozygote deficiency (Table 1), which may be attributed to the Wahlund effect and population not being in HWE.

### Genetic structure, differentiation and migration

**Figure 1:**
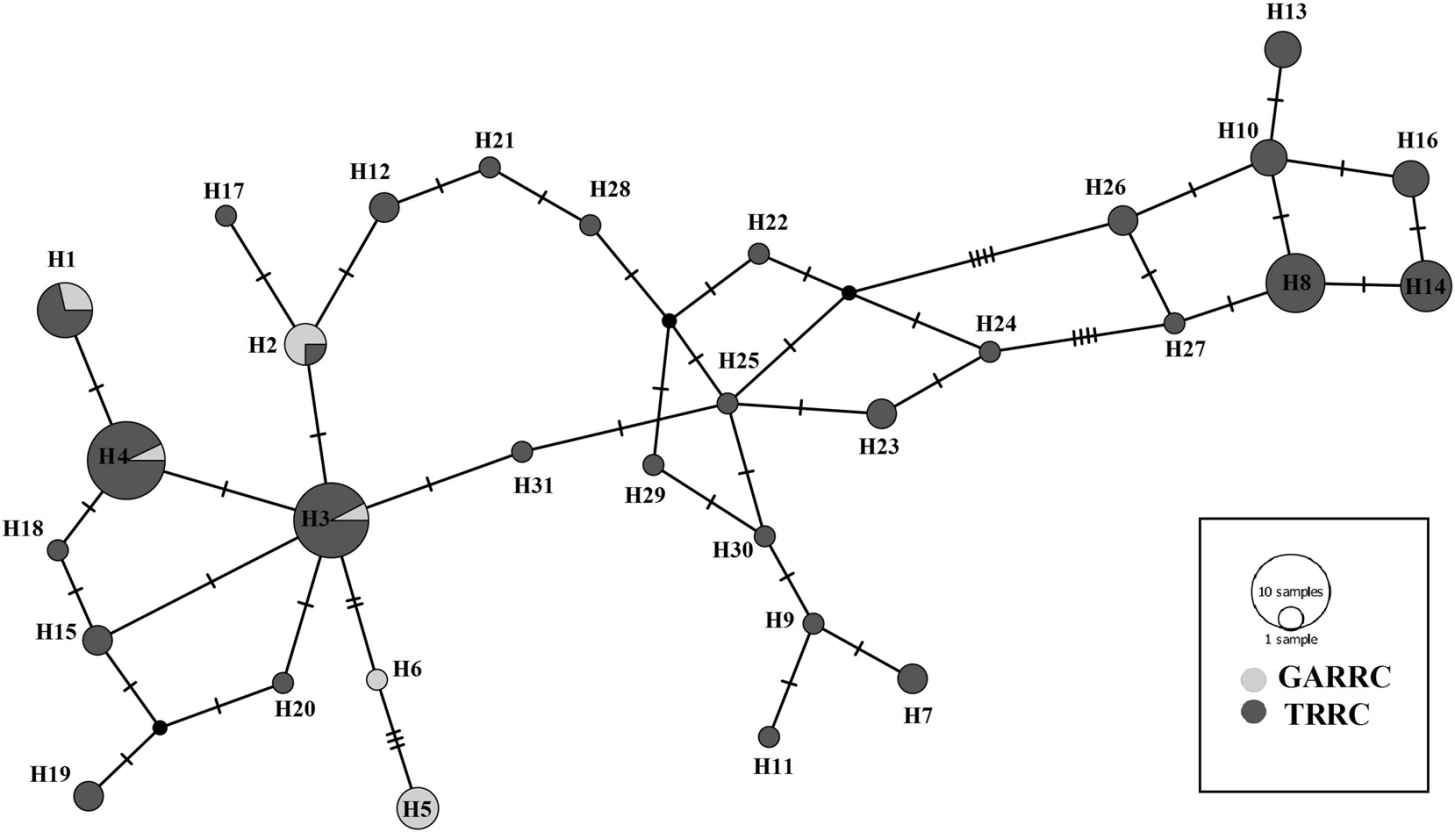
Median-joining network inferred from *Batagur dhongoka* mitochondrial DNA haplotypes. The size of each circle indicates the relative frequency of the corresponding haplotype in the whole dataset. Short tick lines between haplotypes show the number of mutations.

**Figure 2:**
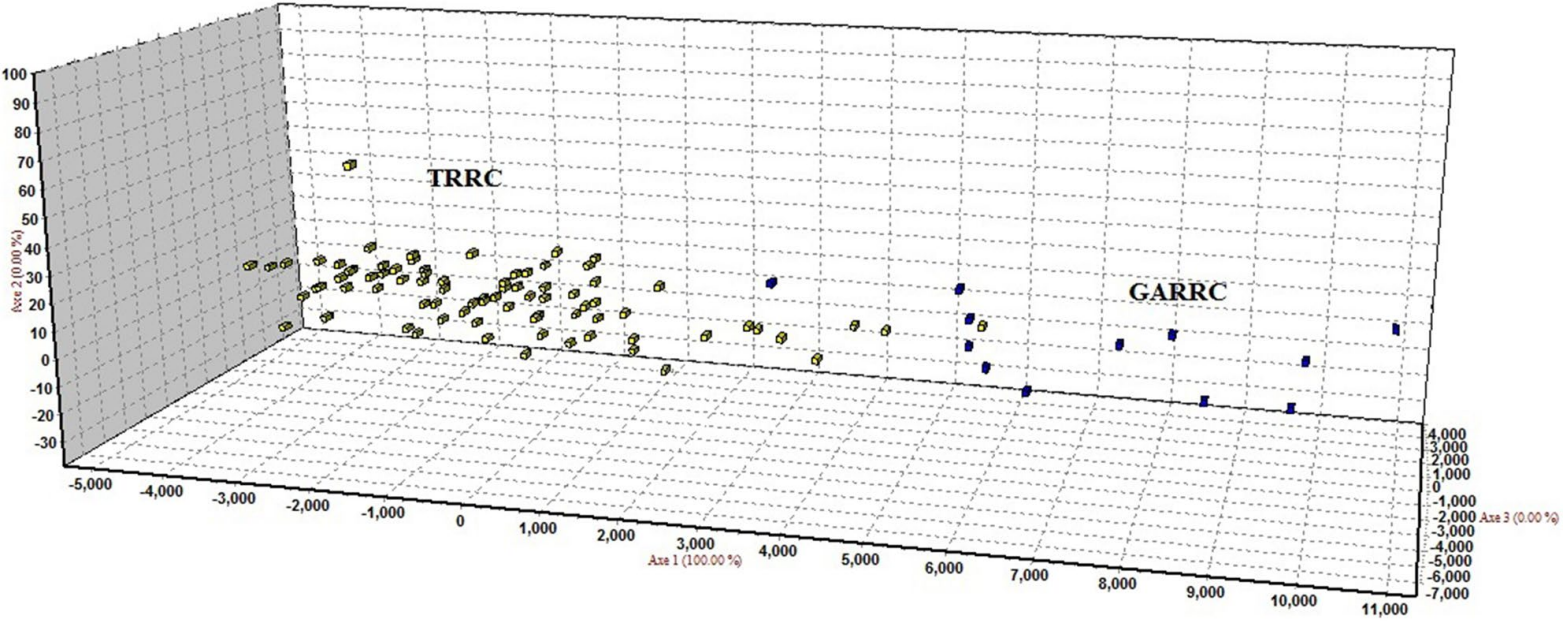
Factorial correspondence analysis (FCA), computed using GENETIX 4.02, shows relationships among the multilocus genotypes of two *Batagur dhongoka* populations (TRRC: Turtle Rescue and Rehabilitation Centre, Sarnath; GARRC: Ganga Aqualife Rescue and Rehabilitation Centre, Narora). Axis 1, Axis 2 and Axis 3 are the first, second and third principal factors of variability.

**Figure 3:**
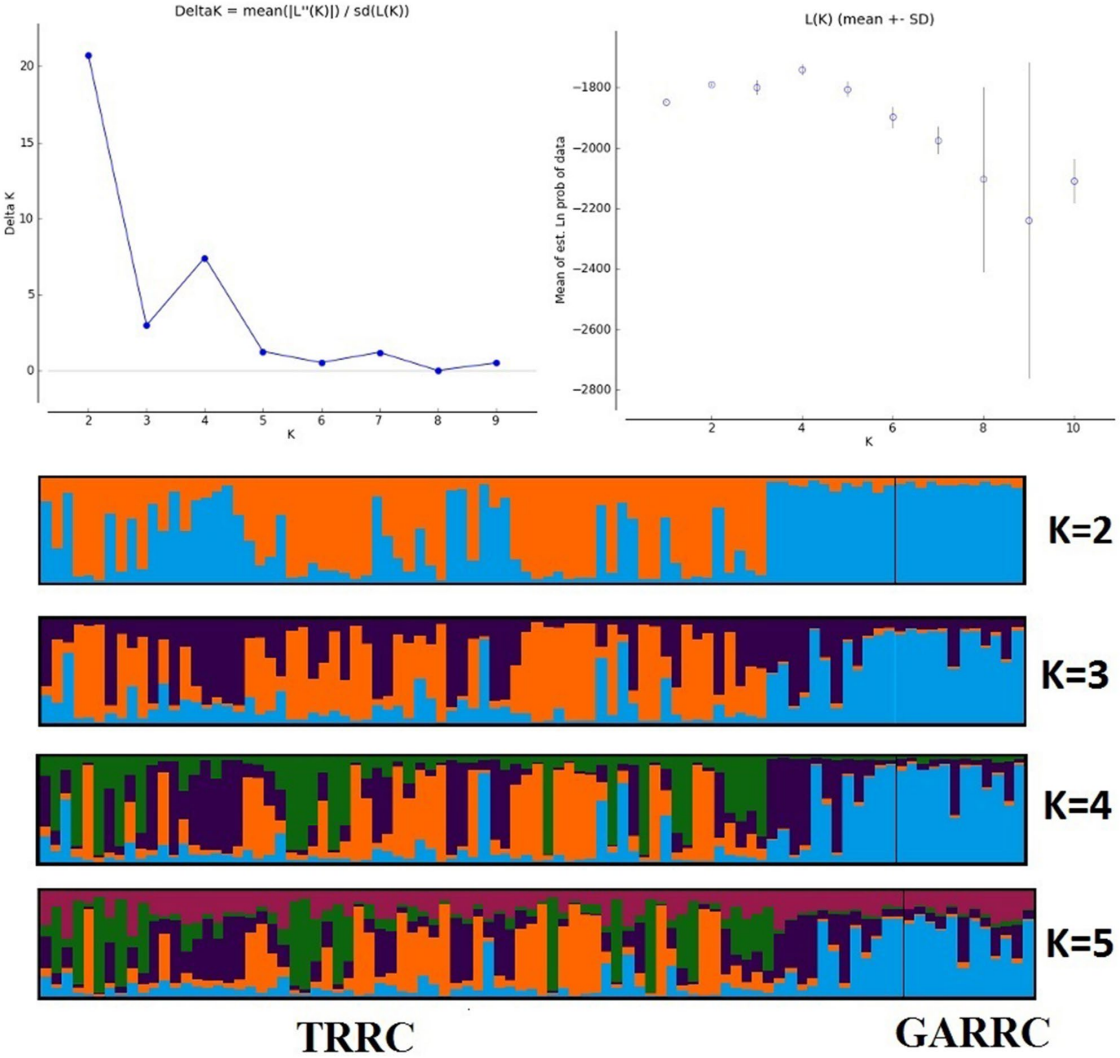
CLUMPK genetic structure plot derived from Bayesian-based cluster analysis in STRUCTURE. The proportion of color in each bar represents the assignment probability of an individual, corresponding to different groups of TRRC: Turtle Rescue and Rehabilitation Centre, Sarnath and GARRC: Ganga Aqualife Rescue and Rehabilitation Centre, Narora.

The median-joining (MJ) network of 31 haplotypes revealed no major branching events among the *B. dhongoka* (Fig. 1). The haplotypes genealogy showed four shared haplotypes (H1- H4), which consists of 38 individuals, representing 41% of total studies populations, out of which H3 and H4 represented the core haplotypes. In TRRC, the majority of the haplotype contained a single sequence. We performed the FCA (Fig. 2) and Structure analysis (Fig. 3) of individual microsatellite genotypes. The sampling plot in the FCA grouped individuals into two suggestive clusters: two groups present in TRRC, whereas GARRC showed a single cluster, and the Bayesian model-based clustering analysis also supported this result. The Bayesian clustering analysis under the admixture model implemented in Structure suggested the existence of two genetic clusters based on high ΔK (mean likelihood of K (Mean LnP(K) =  − 1787.306). The two identified genetic clusters showed some level of genetic admixture and were not assigned to their populations (cluster with an estimated membership > 0.800). The two genetic signature observed in TRRC indicates that the individuals of TRRC population shared a similar genetic ancestry with the GARRC population.

The genetic differentiation level between the studied populations of *B. dhongoka* was lower in mtDNA: F_ST_ = 0.003, whereas a comparatively higher value was observed based on microsatellite: F_ST_ = 0.074. Furthermore, the rate of self-distribution within the GARRC population was 0.9649 (95% CI 0.8730–0.999), while the TRRC population was 0.9413 (95% CI 0.8913–0.9808). The analysis for migration rate (m) was low 0.15 from GARRC to TRRC and 0.035 from TRRC to GARRC.

#### Population demography

**Figure 4:**
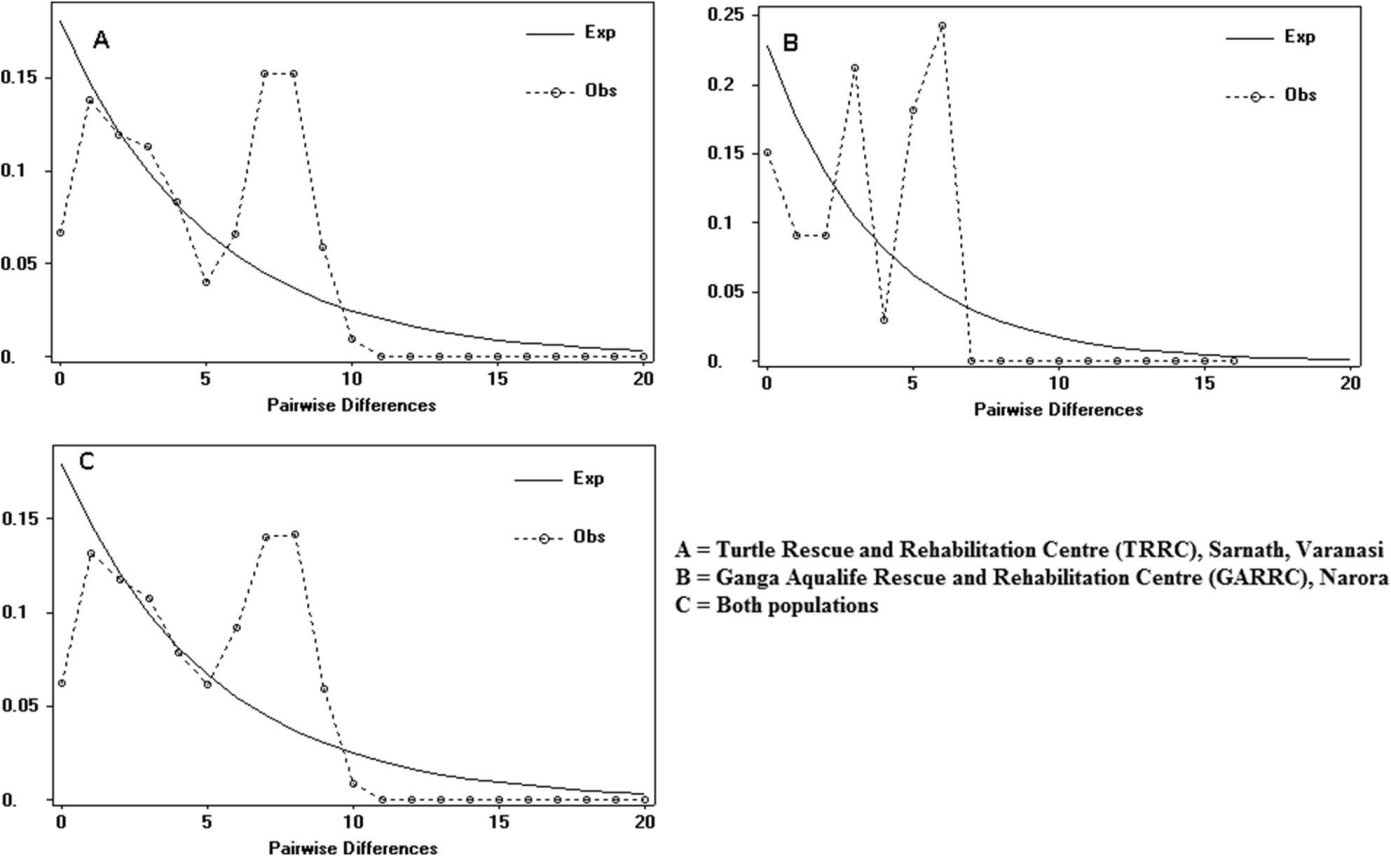
The mismatch distributions graph dashed line showing observed distribution; solid line showing the theoretical expected distribution under a growth-decline model.

**Figure 5:**
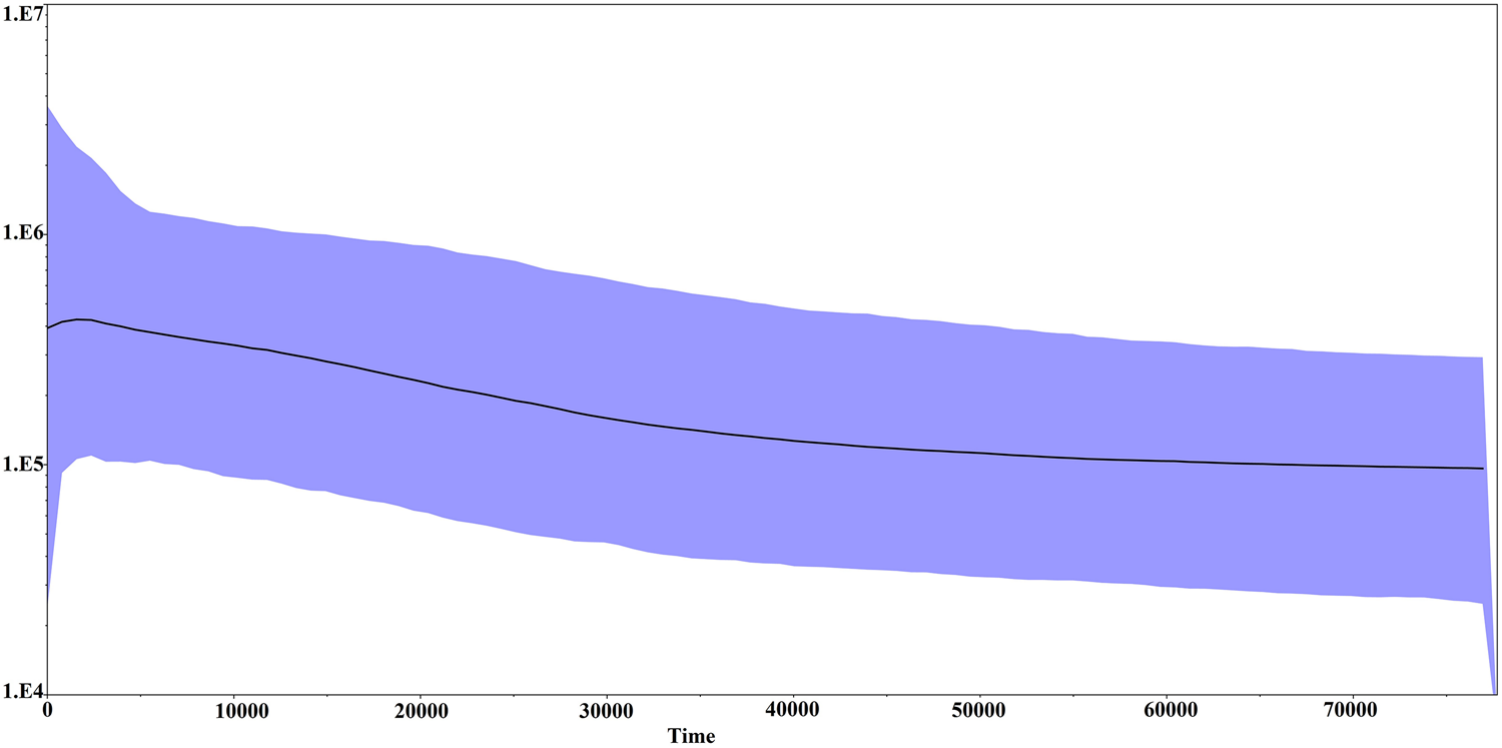
Bayesian skyline plots representing historical demographic trends of *Batagur dhongoka*. X-axis shows time before present in million years ago (MYA). Y-axis indicates effective population size on a logarithmic scale. The thick solid lines represent mean estimates for effective population size, and differentially colored areas reflect the 95% highest posterior density intervals.

The demographic pattern of studied *B. dhongoka* was inferred from neutrality and mismatch distributions analysis. The mismatch distribution analysis showed a multimodal distribution shaped graph (Fig. 4), which might result from a relatively stable population size over the long period. Results of Tajima’s D and Fu’s Fs tests were used to explain the population history of both populations (Table 1). In overall samples, the value Tajima’s D and Fu’s F_S_ was 1.56 and − 1.864, respectively, but non-significant indicated stable population size. Moreover, effective population sizes and demographic trends were also estimated by the Bayesian skyline plot (BSP) analysis. The Bayesian Skyline Plot indicated historical stability followed by growth in the effective population size, with a recent reduction since 2 thousand years (Fig. 5).

#### Bottleneck

The values of G-W index test (TRRC = 0.31 ± 0.07 and GARRC = 0.32 ± 0.11) were lower than the critical value Mc = 0.68, indicating the sign of genetic bottleneck in both the population of *B. dhongoka*. In contrast, BOTTLENECK analysis showed that no significant heterozygosity excess was found in both populations under either TPM (TRRC *P* = 0.72; GARRC *P* = 0.90) or SMM (TRRC *P* = 0.21; GARRC *P* = 0.24). The mode-shift test demonstrated no distortion of the allelic frequency, and a normal L-shaped distribution was observed.

## Discussion

To date, genetic studies of *B. dhongoka* have been limited. Our findings reveal new genetic insight about *B. dhongoka* and thus play an important role in freshwater turtle conservation. This study uses mitochondrial and microsatellite markers to investigate the pattern of genetic diversity, population structure and demography of critically endangered *B. dhongoka* from two turtle rescue and rehabilitation centers, TRRC and GARRC. The analysis of mtDNA revealed high haplotype diversity in concurrence with low nucleotide diversity, indicating that haplotypes were closely related. The possible explanation for turtles' low nucleotide diversity is their slow mitochondrial evolutionary rate^19^. It was also evident from the haplotype network, which shows mostly single nucleotide differences between haplotypes. Similarly, high haplotype diversity have been reported in Loggerhead sea turtle^20^, whereas lower haplotype diversity in turtle species, e.g., in Leatherback turtles, *Dermochelys coriacea*^21^ and Bog turtle, *Glyptemys muhlenbergii*^22^. Our data show a low nucleotide diversity with high haplotype diversity, which can be a signature of population expansion from a small effective population size^23^. The clustering of sequences showed that four haplotypes of TRRC was similar to those found in GARRC, indicating close genetic relationships and having common genetic ancestry. Furthermore, microsatellite analysis revealed a moderate level of genetic diversity for both *B. dhongoka* populations. Microsatellite based structure analysis revealed weak genetic clusters between the TRRC and GARRC populations. While the optimal number of clusters for both populations was determined to be two, we extended our analysis to include K values ranging from 2 to 5. Notably, at K higher than three, the TRRC population showed the presence of two genetic groups, while the GARRC population maintained its unity as a single cluster. The genetic clustering observed in TRRC is probably due to a substantial portion of individuals sharing a similar genetic ancestry with the GARRC population. The results support the ongoing conservation practices under the National Mission for Clean Ganga Project, where most of *B. dhongoka* individuals in TRRC belong to the source population of the Chambal River and few rescued individuals from seizures near the Ganga river. Hence, two different stocks are observed in TRRC. In contrast, individuals in GARRC are only the rescue cases recovered from the local areas.

The BayesAss analysis indicated recent migration from GARRC to TRRC (m = 0.15), which may be attributed to human interference and the mixing of individuals in TRRC (Ganga and Chambal rivers). The rate of self-assemblage was higher in both GARRC and TRRC, which may be due to limited gene flow and low population size. In the current study, our sample size from GARRC is limited. Therefore, it is important to exercise caution when interpreting the conclusions drawn from the BayesAss analysis. Incorporating a larger number of samples along with additional markers has the potential to yield significantly different estimates of migration^24^. Moreover, the results of migration rates were consistent with pairwise F_ST_ values obtained from microsatellite and mtDNA markers, indicating weak genetic differentiation between both the *B. dhongoka* populations. Although *B. dhongoka* has experienced a severe reduction in population size due to anthropogenic disturbance^3^, the mtDNA-based demographic history analyses ruled out any significant past contraction in both the studied populations. It was also in accordance with the estimates of the BOTTLENECK analysis with respect to recent bottlenecks.

In contrast, the G-W index test did not give a consistent result with bottlenecks and mtDNA analysis, suggesting a genetic bottleneck in both populations. It may be accounted for by the G-W index's sensitivity in detecting population bottlenecks, in which the number of alleles is typically more reduced than the range due to a decline in population size^25,26^. As indicated by the Bayesian Skyline Plot, there was a period of historical stability followed by an expansion in the effective population size, but more recently, a slight decline has been observed from ~ 2 thousand years ago. The rise of anthropogenic activities in the Holocene is one of the major factors influencing global species biodiversity^1^. Additionally, the population of *B. dhongoka* has experienced substantial declines due to entanglement in fishing nets, the construction of major hydrological projects affecting river flow dynamics and nesting beaches, water pollution, as well as the detrimental effects of illegal trade^3,5^. We believe these are the most important factors in reducing population sizes and shaping the genetic structure of *B. dhongoka*. Our analysis of microsatellite markers indicates that *B. dhongoka* populations have moderate genetic diversity, while mtDNA analysis suggests a low level of nucleotide diversity. The primary factors contributing to reduced genetic diversity are small population sizes, which are intricately connected to the fitness and health of individuals, along with limited population growth capacity^27^. Therefore, robust rehabilitation and relocation strategies are needed to support small and declining populations, which include scientifically based releases into natural environments. Finding reliable origin information is often challenging, and releasing rescued individuals of unknown sources into the wild is sometimes associated with the mortality of individuals^12,13^. Additionally, while reintroduction starts with a small population, genetic monitoring is essential to comprehend current genetic diversity and relatedness for management and conservation considerations. As a result of the recent trend of population decline amidst persistent pressures, long-term evidence-based conservation, and management interventions are required for the long-term survival of *B. dhongoka*.

### Conservation implications

This study presented a pioneer genetic analysis of *B. dhongoka*. These results expand our genetic knowledge of the Critically Endangered *B. dhongoka* and are significant to support and develop management strategies to manage its genetic diversity in the future. The conservation management of *B. dhongoka* should focus on protecting its core habitats and breeding grounds while tackling illegal poaching and hunting. Its habitat should be monitored and maintained continuously without disturbance and reconnected across its range to maintain a relatively large and healthy effective population size and augment its chances for successful survival and adaptation. If implemented, these measures will conserve not only the *B. dhongoka* but also many threatened sympatric turtle species throughout the Ganga river basin.

## Materials and methods

### Research permits and ethical considerations

The Uttar Pradesh Forest Department vide Letter No. 2036, 3263 and 1854/23-2-12-G, provided necessary permissions for the survey and collection of the biological samples and Institutional Animal Ethics Committee (Letter No. WII/IAEC/2017-18) approved the sampling protocols and has no objection to carrying out the research. The biological samples were collected during 2017 to 2021 as part of a sponsored project under Biodiversity Conservation and Ganga Rejuvenation (Grant no. B-02/2015-16/1259/NMCG-WII-proposal) funded by National Mission for Clean Ganga (NMCG), Ministry of Jal Shakti, Government of India.

#### Sample collection and DNA extraction

**Figure 6:**
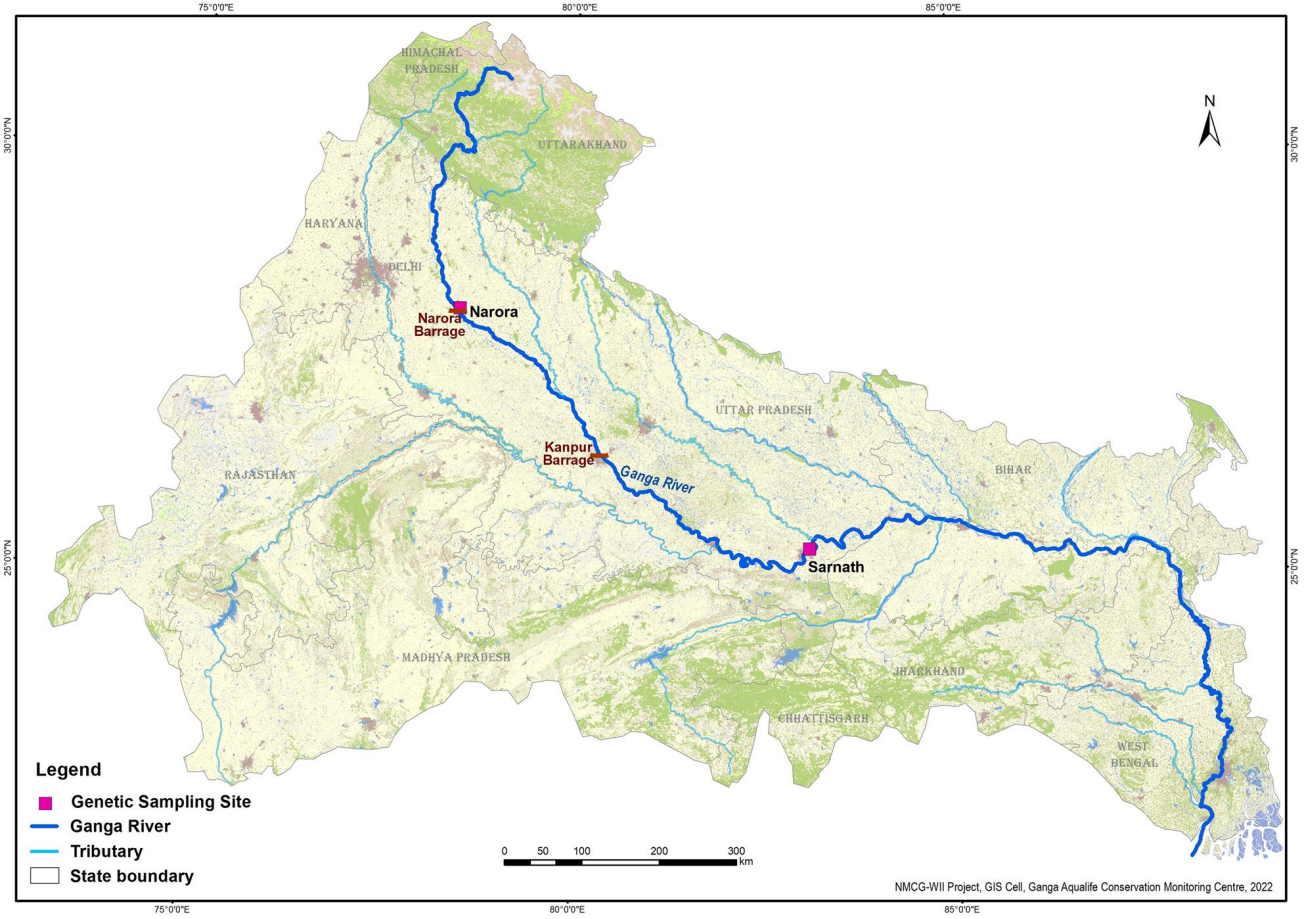
Map of the study area showing sampling sites of *Batagur dhongoka,* which was created using ArcGIS Desktop: Release 10.6.1 Redlands, CA: Environmental systems Research Institute.

A total of 92 tissue samples (eggshell, swabs from hatchlings and remains of dead individuals) were collected from TRRC, Sarnath, Varanasi (n = 80) and GARRC, Narora (n = 12) (Fig. 6). These tissue samples were preserved in 70% ethanol at room temperature. Both the centers, TRRC and GARRC were established near the Ganga river under the aegis of the project Biodiversity Conservation and Ganga Rejuvenation to provide head-start facilities for turtle conservation intended for release into its natural habitat to aid local species recovery. The distance between both centers is approximately 700 km. The TRRC receives turtle eggs from the Chambal river from the Uttar Pradesh forest department and hatched inside the center. Additionally, rescue cases of turtles were also separately maintained in TRRC and GARRC. The genomic DNA (gDNA) was extracted using the DNeasy Blood Tissue Kit (QIAGEN, Germany) in a final elution volume of 100 µl. The gDNA quantification of samples was done in Qubit 4 flurometer (Invitrogen) and diluted to make 50–100 ng/µl for working stock.

### mtDNA amplification and sequencing

Two mitochondrial regions: cytochrome *b* (Cyt *b*) gene ~ 1140 bp and mtDNA control region (CR) ~ 451 bp were amplified using primers: CytbG: 5′–AACCATCGTTGTWATCAACTAC–3′^28^ and mt-f-na3:5′–AGGGTGGAGTCTTCAGTTTTTGGTTTACAAGACCAATG–3′^29^ and CRF: 5′–AGCACCGGTCTTGTAAACCA–3′ and CRR: 5′–ACAAAACAACCAAAGGCCAG–3′^30^. PCR reactions for both regions were performed in 10 µl reaction volumes using 1 × PCR buffer (10 mM Tris–HCl, pH8.3, and 50 mM KCl), 1.5 mM MgCl_2_, 0.2 mM of each dNTPs, 3 pmol of each primer, 5 units of Dream Taq DNA Polymerase (Thermo Scientific) and 1 µl (~ 50 to 100 ng) of template DNA. The PCR protocol was used as follows: initial denaturation at 95 °C for 5 min, followed by 35 cycles of denaturation at 95 °C for 45 s, annealing at 56 °C for Cyt*b* gene and 54°Cfor CR for 60 s and extension at 72 °C for 1 min 30 s. The final extension was at 72 °C for 10 min. Negative controls were included in each reaction to check the reliability and contamination of the experiment. All PCR amplification was confirmed by electrophoresis on 2% agarose gel stained with Firefly dye and visualized under a UV transilluminator. The positive PCR products were further purified with exonuclease-I and shrimp alkaline phosphatase (Thermo Scientific Inc.) at 37 °C for 20 min for the removal of any remaining primer and dNTPs, which was followed by inactivation of enzymes at 85 °C for 15 min. Amplicons were further purified and concentrated via the ethanol precipitation method. The purified fragments were sequenced in both directions in an Applied Biosystems (ABI) Genetic Analyzer 3500 XL (Applied Biosystems) using BigDye version 3.1 Kit.

#### Microsatellite genotyping

Genotyping were performed using thirteen microsatellites loci, including Maucas01, Maucas03, Maucas06, Maucas18, Maucas21, Maucas24^31^; TWT113, TWS190^32^; GP19^33^; GmuD16, GmuB08^22^; Test 21^34^; msEo41^35^. Of these, three ‘Maucas21, Maucas24 and GmuD16’ did not amplify consistently in all samples; hence, excluded from the further analysis. Therefore, final analysis was performed with ten microsatellite markers. The forward primer of each marker was attached with one of the 5′ universal sequence tail: T7 (5′-TAATACGACTCACTATAGGG), M13 (5′-TGTAAAACGACGGCCAGT) and M13R (5′-CAGGAAACAGCTATGACC)^36^. The universal primers M13, M13R and T7 were labelled with fluorescent dye FAM, HEX, and TET, respectively. All fluorescent-labelled universal primer were dissolved in Tris EDTA buffer (10 mM Tris, 1 mM EDTA, pH 8.0) to produce a 20 mM stock solution and 5 mM locus-specific forward and reverse primer. Primer mix was prepared with universal tail labelled forward primer: reverse primer: dye-labelled universal primer = 1:4:4^36^. Multiplexing of primers was carried out according to fragment size and dye-labelled (Supplementary table ST2). PCR was carried out in 10 µl PCR reaction volumes, containing 5 µl of QIAGEN Multiplex PCR Buffer Mix (QIAGEN Inc.), 0.4 µM primer mix, and 80–100 ng of the template DNA. The PCR cycle was performed under the touchdown conditions, initial denaturation at 95 °C for 15 min, followed by 8 cycles of denaturation at 95 °C for 45 s, annealing at 58 °C for 60 s and extension at 72 °C for 75 s, again followed by 15 cycles of denaturation at 95 °C for 45 s, annealing at 58–50 °C for 60 s and extension at 72 °C for 75 s which followed by 12 cycles of denaturation at 95 °C for 45 s, annealing at 52 °C for 60 s and extension at 72 °C for 75 s with final extension of 60 °C for 30 min. Allelic sizes were scored against the size standard GS500 LIZ. We visualized amplicons using an ABI 3500 XL automated sequencer. Genotypes were identified using GeneMarker version 2.7.4 (Applied Biosystems, Foster City, California, USA).

### Data analysis

#### Mitochondrial DNA diversity

The forward and reverse sequences were aligned and edited using SEQUENCHER version 4.9 (Gene Codes Corporation, AnnArbor, MI, USA). All the sequences were aligned using CLUSTAL W^37^, and checked manually in BioEdit v 7.2.5 software^38^. DnaSP 5.0^39^ was used to analyze the haplotype diversity (h), nucleotide diversity(p), polymorphic sites (s) and mean number of pairwise differences among sequences (K). MEGA X^40^ was used to calculate mean within and between population genetic distances (uncorrected p-distances).

#### Population demography

The spatial distribution of the haplotypes was visualized through a median-joining network, which was created using the PopART software^41^. To determine whether the *B. dhongoka* populations carried a signal of demographic expansion or astationary population history, Tajima’s D^42^ and Fu’s Fs^43^ neutrality tests were performed in Arlequin ver 3.5^44^. To generate the trends in spatial demography history, mismatch analysis was carried out using the population growth-decline model in DnaSP^39^, and to evaluate fit of the observed distribution, sum of squared deviations (SSD), and raggedness index(r) under the growth-decline model for each population were used in Arlequin ver 3.5^44^. The P-values were obtained from 1000 simulations based on a selective neutrality test. Best-fit nucleotide substitution model was selected based on Akaike information criterion (AIC) and Bayesian information criterion (BIC) using jModel Test v2.1.7^45^. Bayesian skyline analyses available in BEAST v1.7^46^ were used to infer changes in effective population size (Ne) across time, enabling inference of past demographic changes from the current patterns of genetic diversity within a population^47^. We performed multiple analyses that were run for 10^8^ iterations with a burn-in of 10^7^ under the TN93 + G model. Because no fossil data were available to calibrate the mutation rate, we assume a conventional molecular clock for the turtle mitochondrial gene (1.75 × 10^−8^ substitutions/site/year^48,49^). The mean mutation rate was also consistent with the other turtle species mutation rate of 1.2–2.4%/site per Myrs^50^. Length of MCMC chain was 100 million and sampling every 10,000 generations. Results of replicate runs were combined using LogCombiner v1.7.5^46^ with a burn-in of 20 million iterations (20%) for each run. Tracer v1.7.1^51^ was used to assess the convergence of chains and to reconstruct Bayesian Skyline Plot.

#### Microsatellite analysis

Microsatellite data was analyzed using the program Micro-Checker 2.2^52^ to identify genotyping errors, such as null alleles, large allele dropout, or errors in scoring. The number of effective alleles (Ne), alleles per locus (Na), observed heterozygosity (Ho), and expected heterozygosity (H_E_) were estimated using the GenAlEx v6.5 program^53^. The polymorphic information content (PIC) values were calculated using CERVUSv3.0.7^54^. The supporting format of the data input files was prepared using CONVERTv1.31^55^. Allelic richness (Ar) and Inbreeding (FIS) were calculated in Fstat 2.9.3.2 for each population, and its significance was tested, assuming no Hardy–Weinberg equilibrium within the samples and using 1000 permutations^56^. All the loci were checked for under HWE in GenAlEx v6^53^. To determine the level of distortion from independent segregation of loci, Linkage disequilibrium (LD) was tested using GENEPOP on the web (genepop.curtin.edu.au).

#### Genetic structure, differentiation and migration

The pairwise F_ST_ values (gene flow) among the populations were calculated using GenAlEx v6^53^. Initially, a factorial correspondence analysis (FCA) was performed using GENETIX ver. 4.02^57^, which graphically represents the genetic distances between individual multilocus genotypes. Finally, we tested the population genetic structure using the Bayesian method using Structure version 2.3.3^58^. We assumed an admixture model with correlated allele frequencies with a burn-in period of 60,000 and 6,00,000 MCMC repetitions. Twenty independent replicates were run for K = 1–10, without prior knowledge of sampling locations. We determined the optimal value of K according to the ΔK method^59^ using web server of Structure Harvester^60^. The clustering results of Structure were visualized using a web server ClumpaK (http://clumpak.tau.ac.il/index.html) that provides a full pipeline for clustering, summarizing, and visualizing Structure results. We used BayesAss v.1.3 program to estimate recent migration rates, m (over the past few generations) among populations based on multilocus genotypes using Markov chain Monte Carlo^61^. We used burn-in iterations 10^6^ followed by 10^7^ iterations and a sampling frequency of 2000. The initial run was performed with the default delta (Δ) value of 0.15 for allele frequencies (A), migration (M), and inbreeding coefficient (F). The final input parameter of ΔM was adjusted at 0.2. As recommended in the manual the changes in these parameters would be accepted between 40 and 60% of the total number of iterations. Four independent runs were also performed to validate the consistency of the results.

#### Population history: bottlenecks

We used Garza–Williamson (G-W) index^62^ and BOTTLENECK v.1.2.02^63^ programs to determine the signal of past bottlenecks. We estimated G-W index using ARLEQUIN v3.5^44^, which is a mean ratio of the number of observed alleles in a sample divided by the number of alleles expected under the observed size range and can detect population bottlenecks from the past^62^. The value of *M* ratio below the critical *Mc* value of 0.68 was considered a sign of a genetic bottleneck. Second, BOTTLENECK program was run under two mutation models: two-phase model (TPM) and the stepwise mutation model (SMM). The proportion of alleles attributed to SMM under the TPM was set at 90%, with a variance of 12. Significance was determined by the sign and Wilcoxon tests with 10,000 replications^63^. We also checked allele frequency distributions for mode shifts that discriminate populations that have experienced a recent bottleneck from stable populations^64^.

### Ethical approval and informed consent

This study did not involve human clinical data; hence no informed consent was required for this work. Samples were mostly collected from the dead remain and necessary permission has been obtained from the forest department. All methods were carried out in accordance with relevant guidelines and regulations. This study was carried out in compliance with the ARRIVE guidelines. The experiments were conducted after obtaining necessary approval from the Institutional Animal Ethics Committee vide letter no. No. WII/IAEC/2017-18 dated 25/06/2020.

### Supplementary Information

Supplementary Information.

## Data Availability

All the relevant sequencing data has been uploaded on NCBI, GenBank (OQ378390–OQ378573).
