# Supplementary material for: Population genetics of the critically endangered three-striped turtle, Batagur dhongoka, from the Ganga river system using mitochondrial DNA and microsatellite analysis
Source: Sci Rep. 2024 Mar 11;14:5920. doi: 10.1038/s41598-024-54816-0 (PMC10928089; doi:10.1038/s41598-024-54816-0)
Supplement: Supplementary file 1 — Supplementary Information. [file 41598_2024_54816_MOESM1_ESM.doc]

**Population genetics of the critically endangered Three-Striped turtle, *Batagur dhongoka*, from the Ganga river based on mitochondrial and microsatellite analysis**

**Supplementary Table ST1: Summary of genetic diversity of *Batagur dhongoka* inferred from 10 microsatellite markers.**

|  | **All Populations (n=92)** | | | | | | **TRRC (n=80)** | | | **GARRC (n=12)** | | |
| --- | --- | --- | --- | --- | --- | --- | --- | --- | --- | --- | --- | --- |
| **Loci** | **Range** | **Na** | **Ne** | **Ho** | **He** | **F** | **Na** | **Ho** | **He** | **Na** | **Ho** | **He** |
| Maucas01 | 158-176 | 5 | 1.31 | 0.170 | 0.239 | 0.283 | 5 | 0.114 | 0.200 | 2 | 0.650 | 0.660 |
| Maucas03 | 191-195 | 2 | 1.22 | 0.205 | 0.185 | -0.114 | 2 | 0.228 | 0.202 | 1 | 0 | 0 |
| Maucas06 | 152-184 | 6 | 3.12 | 0.753 | 0.680 | -0.107 | 6 | 0.763 | 0.674 | 4 | 0.620 | 0.685 |
| Maucas18 | 270-318 | 12 | 7.74 | 0.767 | 0.876 | 0.119 | 12 | 0.740 | 0.867 | 9 | 0.820 | 0.864 |
| TWT113 | 254-260 | 3 | 1.75 | 0.618 | 0.430 | -0.436 | 3 | 0.575 | 0.413 | 2 | 0.840 | 0.500 |
| TWS190 | 102-118 | 6 | 2.73 | 0.739 | 0.635 | -0.164 | 6 | 0.709 | 0.599 | 5 | 0.870 | 0.704 |
| Gmub08 | 212-230 | 7 | 3.72 | 0.448 | 0.731 | 0.389 | 6 | 0.487 | 0.750 | 3 | 0.300 | 0.450 |
| GP19 | 256-264 | 3 | 1.24 | 0.124 | 0.196 | 0.369 | 3 | 0.125 | 0.163 | 3 | 0.111 | 0.640 |
| Test21 | 218-232 | 4 | 2.15 | 0.391 | 0.535 | 0.270 | 4 | 0.420 | 0.556 | 1 | 0 | 0 |
| MsE041 | 97-106 | 3 | 2.02 | 0.742 | 0.508 | -0.461 | 3 | 0.713 | 0.506 | 2 | 0.717 | 0.500 |
| **Mean** |  | **5.1** | **2.70** | **0.496** | **0.501** | **0.051** |  | **0.487** | **0.493** |  | **0.497** | **0.500** |

*Na* number of different alleles, *Ne* Number of effective alleles, *Ho* observed, heterozygosity, *He* expected heterozygosity, *F* Fixation Index. TRRC: Turtle Rescue and Rehabilitation Centre, Sarnath, Varanasi; GARRC: Ganga Aqualife Rescue and Rehabilitation Centre, Narora.

**Supplementary Table ST2: List of primers and its multiplexing performed** in the study

| **Primer ID** | **Primer Sequence with 5′ universal sequence tail** | **Labeling** | **Adapter** | **Multiplexing/Pool** | **Reference** |
| --- | --- | --- | --- | --- | --- |
| Maucas01F | TAATACGACTCACTATAGGGTGAATAGATAGAGGGAGTGTCTGG | TET | T7 | I | Vamberger et al., 2011 |
| Maucas01R | TGAACCTGCGAAAATACTGC |
| Maucas03F | TAATACGACTCACTATAGGGCCTTCCCCATTGATTAAATGTC | TET | T7 | II | Vamberger et al., 2011 |
| Maucas03R | TTGGAGGCAAAGGATGAGAC |
| Maucas06F | TGTAAAACGACGGCCAGTGAGACCTGCACCCAAATCAT | FAM | M13 | III | Vamberger et al., 2011 |
| Maucas06R | TGTGCAAAGTGTGTGGAGATT |
| Maucas18F | CAGGAAACAGCTATGACCACATTGGTCACCATGCAGAG | HEX | M13R | I | Vamberger et al., 2011 |
| Maucas18R | GGGACTGAAAACCAAGATGC |
| TWT113F | TAATACGACTCACTATAGGGCTTTTAGGCTGGGCTGATTG | TET | T7 | II | Perez et al., 2006 |
| TWT113R | ATGCAACCCCAGTACCTCTG |
| TWS190F | CAGGAAACAGCTATGACCTTGTTCTGCCATCAGTCAGC | HEX | M13R | I | Perez et al., 2006 |
| TWS190R | ATCCCCTTACCACCAACTCC |
| GP19F | TGTAAAACGACGGCCAGTGCAGGACAGTGCCACACTA | FAM | M13 | III | Schwartz et al., 2003 |
| GP19R | CAGCCATATTAATGACAATCTG |
| GmuB08F | TAATACGACTCACTATAGGGCTCTGAGACCCTTAT TCACGTC | TET | T7 | III | King et al., 2004 |
| GmuB08R | AGCCTTTGTCTGTAAGCTGTTC |
| Test 21F | TGTAAAACGACGGCCAGTAAACTGGCTGAAACCCAGC | FAM | M13 | II | Forlani et al., 2005 |
| Test 21R | TTGGGAGTTTGACTGATCTAGGA |
| msEo41F | TGTAAAACGACGGCCAGTATAGCTTCAGCCTTAACTGTG | FAM | M13 | II | Pedall et al., 2009 |
| msEo41R | AGCCAGAACTATGGGGGTG |
